# Diagnostic performance of C-TIRADS combined with SWE for the diagnosis of thyroid nodules

**DOI:** 10.3389/fendo.2022.939303

**Published:** 2022-09-06

**Authors:** Xiao-Qiang Gao, Yue Ma, Xiao-Shan Peng, Ling-Ling Wang, Hai-Xia Li, Xiu-Lan Zheng, Ying Liu

**Affiliations:** Department of Ultrasound, Harbin Medical University Cancer Hospital, Harbin, China

**Keywords:** ultrasonography, thyroid nodules, shear wave elastography, TIRADS, thyroid imaging reporting and data system, fine-needle aspiration biopsy

## Abstract

**Objective:**

To explore the value of the optimal parameters of shear wave elastography (SWE) to enhance the identification of benign and malignant thyroid nodules by C-TIRADS.

**Methods:**

The two-dimensional ultrasonography images and SWE images of 515 patients with a total of 586 thyroid nodules were retrospectively analyzed. The nodules were divided into the D ≤10 mm and D >10 mm groups according to size and were graded by C-TIRADS. With the pathological results as the gold standard, the receiver operating characteristic (ROC) curves were drawn, and the area under the curve (AUC) was calculated to compare the diagnostic performances of C-TIRADS, SWE, and the combination of the two on the benign and malignant thyroid nodules.

**Results:**

The ROC showed that the AUC of the maximum elastic modulus (0.875) was higher than that of the mean elastic modulus (0.798) and elasticity ratio (0.772), with an optimal cutoff point of 51 kPa, which was the optimal parameter to distinguish the malignant from the benign nodules (*P* < 0.001). In the D ≤10 mm group, the AUC of TIRADS combined with SWE (0.955) was elevated by 0.172 compared with the application of C-TIRADS alone (0.783), and the difference was statistically significant (*P* < 0.05). In the D >10 mm group, the AUC of TIRADS combined with SWE (0.904) was elevated by 0.076 compared with the application of C-TIRADS alone (0.828), and the difference was statistically significant (*P* < 0.05). Among all nodules, the application of C-TIRADS alone had a sensitivity of 88.14%, a specificity of 74.56%, and an accuracy of 85.50% in diagnosing benign and malignant thyroid nodules, while the sensitivity, specificity, and accuracy were 93.22%, 90.35%, and 92.66%, respectively, in combination with SWE.

**Conclusion:**

The diagnostic performance of SWE in combination with TIRADS was better than that of SWE or C-TIRADS alone. Here, SWE enhanced the diagnostic performance of C-TIRADS for the benign and malignant thyroid nodules, most significantly for nodules with D ≤10 mm.

## Introduction

In recent years, due to the popularity of thyroid-related screening, the number of unpalpable thyroid nodules detected has increased, with an incidence of 50% to 60% (1), causing widespread concern. However, only 7%–15% of thyroid nodules are proven to be malignant, and only a small percentage of malignant nodules require surgery; many benign thyroid nodules and low-risk papillary thyroid microcarcinoma are only recommended for active surveillance (2). Therefore, determining the risk of malignancy of thyroid nodules and deciding on the management of thyroid nodules are primary challenges for ultrasonographers and clinicians. Accurate and reliable diagnostic methods are vital in identifying malignant thyroid nodules and reducing overdiagnosis and overtreatment of benign nodules.

Currently, there are several methods for the identification of benign and malignant thyroid nodules. In addition to conventional imaging techniques, fine-needle aspiration (FNA) and genetic testing are also widely adopted. High-frequency ultrasonography is the first choice of imaging examinations and is widely used because it is non-invasive, low cost, convenient, and non-radioactive among other advantages. However, ultrasonography technology has limitations and deficiencies in differentiating benign and malignant thyroid nodules. The complex and diverse structure of thyroid nodules and the overlap between benign and malignant signs reduce the diagnostic accuracy and specificity. Thus, researchers have conducted extensive investigations to overcome these difficulties and find more effective and accurate diagnostic methods.

To better align the ultrasonography diagnosis of thyroid nodules with the current medical situation in China, the Ultrasound Medicine Branch of the Chinese Medical Association published the “2020 Chinese Guidelines for Ultrasound Risk Stratification of Thyroid Nodules for Malignancy: C-TIRADS” (C-TIRADS Guidelines) in July 2020 (3). C-TIRADS is graded using the counting method. That is, suspicious ultrasound signs are counted, and one point is added for each suspicious sign and one point subtracted in the presence of a comet tail sign. Based on the total score, thyroid nodules are graded for malignancy risk and classified from grades 1–6 as per the risk. Clinicians determine the treatment options based on the nodule grading. This can reduce the biopsy rate of nodules and has great application. However, some nodules have unclear signs of malignancy and may present only as solid and hypoechoic, with no other clear signs. Most of these nodules are graded as TIRADS grade 4, and the existing grading methods have difficulty distinguishing benign properties from malignant ones, which makes management and treatment difficult for clinicians. As an invasive examination, FNA also has its limitations, such as local complications and potentially non-diagnostic and indeterminate results (4) Therefore, in addition to traditional two-dimensional (2D) ultrasonography, researchers are looking for a simple and non-invasive method that can provide physicians with more diagnostic information to help assess the benign or malignant nature of thyroid nodules.

Shear wave elastography (SWE) has developed rapidly in recent years. Due to its ability to accurately assess tissue stiffness, it is widely applied for superficial organs and the liver. The tissue hardness is assessed by SWE based on Young’s modulus. Nodules with a higher hardness are usually at a higher risk of malignancy due to the internal structure. Shear wave elastography may provide important information for identifying benign and malignant nodules. Studies have been conducted to evaluate its diagnostic performance in combination with TIRADS (5–11). However, the combined application of C-TIRADS with SWE has not been evaluated, and some studies have evaluated C-TIRADS alone. Therefore, the adjunctive performance of SWE on C-TIRADS is unclear.

The purpose of the present study is to investigate the diagnostic performance of SWE and C-TIRADS applied alone and to assess the adjunctive diagnostic value of SWE for C-TIRADS in differentiating malignant nodules from benign ones.

## Materials and methods

### Study objects

A total of 515 patients with 586 thyroid nodules detected by ultrasonography who visited our hospital from October 2019 to May 2021 were selected. There were 75 males (89 nodules) and 440 females (497 nodules), with an age range of 24–72 years and an average age of 45.81 ± 8.77 years. The maximum diameter of the nodules ranged from 2 to 60 mm, with an average diameter of 15.07 ± 10.15 mm. All the cases included in this study obtained clear FNA or postoperative pathological results, and the cytological types of some patients whose FNA could not be defined were determined after surgery. A total of 12 cases with unclear pathological results were excluded. The inclusion criteria were as follows: (1) patients >18 years old; (2) patients with no previous biopsy, thyroid surgery, or thermal ablation therapy; (3) patients who underwent conventional ultrasound and SWE examination and had clear FNA results or postoperative pathological results were included in strict accordance with the inclusion and exclusion criteria, excluding those whose data were incomplete or did not meet the requirements.

### Apparatus and methods

A Supersonic Imagine Aixplorer SWE color Doppler ultrasound with an L4-15 line array probe and a frequency setting of 4–15 MHz was adopted. The patient was placed in a supine position with the head dorsiflexed, and multilayer, multi-angle scanning of the thyroid and surrounding area was conducted. As the probe moved, the target node was set in the center of the image, and conventional transverse and longitudinal ultrasonography images of the target node were stored, recording the nodule’s size, location, margins, structure, echoes, and focal strong echoes. Upon completion of the conventional ultrasonography, SWE was conducted. The energy transducer was held perpendicular to the skin without pressure, and the patient was instructed to hold their breath. SWE mode is then enabled, where 2D ultrasound and SWE images were displayed in dual mode, with the SWE image displayed as a multi-colored area. Adjust the area of Region of interest (ROI), which is usually 2-3 times of the lesion. If the lesion is large, part of normal thyroid tissue should be included. Place the nodule in the center of ROI, when the image is stable and there is no artifact the ultrasonographer froze the image and placed the Q-box, Q-box should be placed within the lesion, avoiding cystic or calcified areas, then the operator conducted a quantitative elastography assessment, which included measurement of the maximum elastic modulus (Emax), mean elastic modulus (Emean), and elasticity ratio (Eratio). The examination was repeated three times, and three sets of data were obtained; the average value of the Young’s modulus of these sets was taken as the final result. To reduce measurement errors caused by different operators, all examinations were conducted by the same doctor (who had five years of experience in SWE).

### C-TIRADS grading and the judgment of benign and malignancy

**Figure 1:**
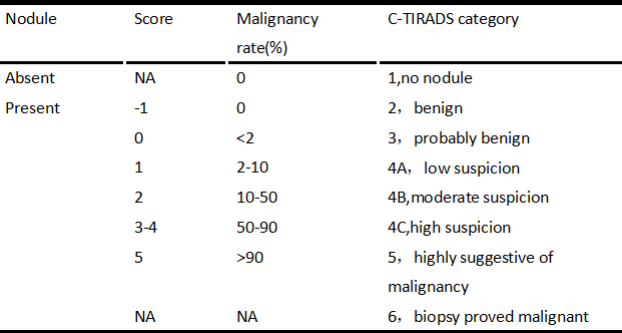
C-TIRADS based on counting method.

**Figure 2:**
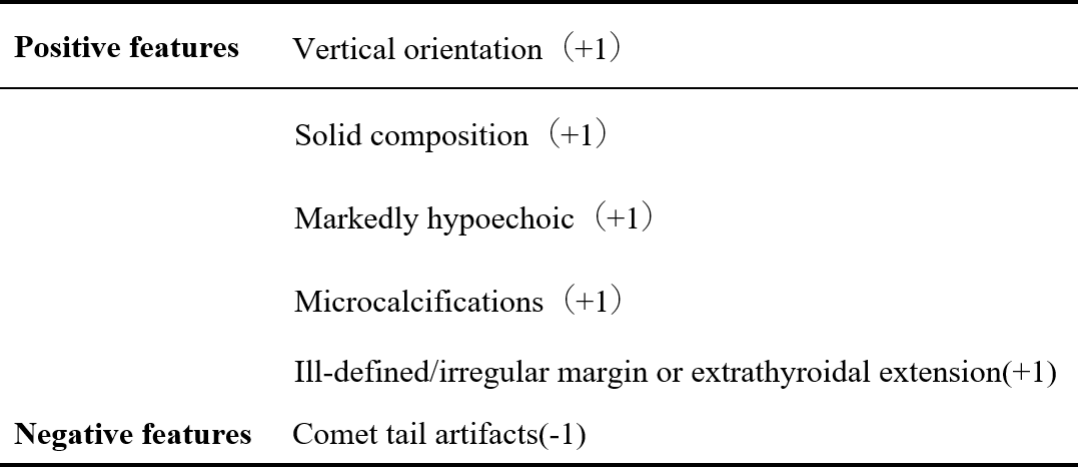
List of suspicious ultrasound features based on C-TIRADS.

**Figure 3:**
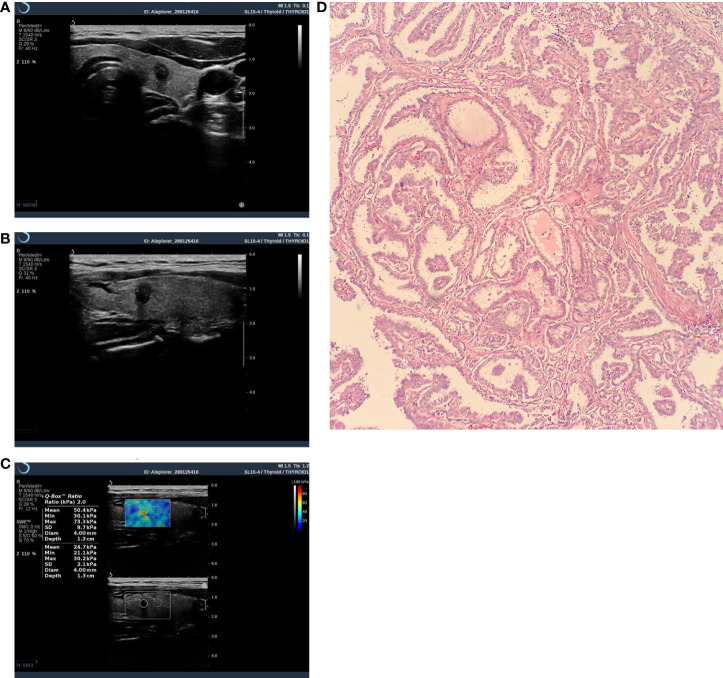
Thyroid nodule image of a 43-year-old female patient. It was diagnosed as malignant by C-TIRADS and SWE. **(A)** Crosscut two-dimensional ultrasound with a C-TIRADS rating of 4c. **(B)** Longitudinally cut two-dimensional ultrasound. **(C)** SWE image, Emax=73.3kpa. **(D)** Pathological image(HE×100),Postoperative histopathological examination showed papillary thyroid carcinoma.

All nodules were graded by two ultrasound specialists according to the grading method in the C-TIRADS guidelines and the specified suspicious ultrasound features (**Figures 1**, **2**). In the case of discrepancy between the specialists on the evaluation of a particular thyroid nodule, a consensus was reached in consultation with a third physician. The criteria for the judgment of benign and malignant nodules were as follows: With the adoption of the optimal diagnostic parameter of SWE, a nodule with an elasticity greater than the optimal cutoff point was determined as a malignant nodule. A nodule with an elasticity lesser than or equal to the optimal cutoff point was determined as a benign one. When SWE was combined with C-TIRADS, the C-TIRADS grading was increased by one level for nodules with an elasticity greater than the optimal cutoff point (as shown in **Figure 3**). For nodules with an elasticity lesser than or equal to the optimal cutoff point, the C-TIRADS grading was decreased by one level. When the nodule grade was ≥4C, the final diagnosis of the nodule was determined to be malignant. When the nodule grade was ≤4B, the final diagnosis of the nodule was determined to be benign; that is, nodules with grades 4C and 5 were malignant nodules, and the rest were benign.

### Statistical methods

The SPSS 22.0 and MedCalc 15.9 statistical software were adopted for statistical analysis. The normal distribution of data was tested by the Shapiro–Wilk test. The measurement data were expressed as the mean ± standard deviation. The independent sample t-test was used for the analysis of the patients’ general characteristics. The χ2 test was adopted for the analysis of the ultrasonography features of the nodules. With the pathological results as the gold standard, the receiver operating characteristic (ROC) curves were drawn to obtain the area under the curve (AUC). The optimal SWE parameter was selected, and the maximum point of the Jordan index of the optimal parameter was adopted as the optimal cutoff point. The ROC curves for the SWE optimal parameters, the application of SWE and TIRADS alone, and the combination of the two methods were plotted to calculate the sensitivity, specificity, and AUC. The DeLong test was used to compare the significance of the differences. A *P*-value <0.05 was considered statistically significant.

## Results

### The general characteristics of the patients and the benign and malignant nodules

A total of 586 thyroid nodules in 515 patients (444 patients with 1 nodule and 71 patients with 2 nodules) were included in the present study. Among the 586 nodules, there were 114 benign ones, including 89 cases with nodular goiter, 17 cases with adenoma, and 8 cases with inflammatory nodules; there were 472 cases with malignant nodules, all of which were papillary carcinomas. Overall, 45 cases with benign nodules and 220 cases with malignant ones were confirmed by surgical examination, and 69 cases with benign nodules and 252 cases with malignant ones were confirmed by cytological examination. In each group, the average age of the patients with malignant nodules was younger than that of the patients with benign nodules (*P* < 0.05 in both), and the difference in gender distribution was not statistically significant between those with benign nodules and those with malignant ones (*P* > 0.05 in all; **Table 1**).

**Table 1:** Size, number of nodules and age of patients.

Parameter	The number of nodules	Patient	Final diagnosis		P
			Benign	Malignant	
All nodules	586	515	114	472	
Age		45.81 ± 8.77	50.68 ± 8.96	44.59 ± 8.27	<0.001^∗^
Female	497	440	91	406	0.133
Male	89	75	23	66	
D ≤ 10mm					
Age		46.01 ± 8.52	49.86 ± 7.61	45.09 ± 8.63	0.006
Female	176	146	24	152	0.770
Male	34	28	4	30	
D>10mm					
Age		45.80 ± 8.81	50.95 ± 9.38	44.28 ± 8.03	<0.001^∗^
Female	321	294	67	254	0.051
Male	55	47	19	36	

(The age in patients was expressed as means ± standard deviations, ∗ indicating a statistically significant difference).

### The ultrasonography features of the nodules

The nodules’ conventional ultrasonography features revealed that the differences in the orientation, margins, composition, echogenicity, punctate echogenic foci, and peripheral calcification were statistically significant between the benign and malignant nodules (P < 0.001 in all), while the difference in the distribution of Macrocalcifications was not (P = 0.707, P > 0.05). Among these ultrasonography features, a vertical orientation (taller-than-wide, the long axis of the nodule and skin line tended to be vertical), ill-defined margins or extrathyroidal invasion, solidity, markedly hypoechoic, and microcalcifications were more common in the malignant nodules and included in the scoring criteria of C-TIRADS (**Table 2**).

**Table 2:** Conventional ultrasound features of benign and malignant nodules.

Features	The number of benign nodules (*n*=114)	The number of malignant nodules (*n*=472)	P
Orientation			<0.001^∗^
Vertical (*n*=367)	35 (30.70%)	332 (70.34%)	
Horizontal (*n*=219)	79 (69.30%)	140 (29.66%)	
Margin			<0.001^∗^
Circumscribed (*n*=138)	58 (50.88%)	80 (16.95%)	
Irregular (*n*=278)	15 (13.16%)	263 (55.72%)	
Ill-defined (*n*=153)	41 (35.96%)	112 (23.73%)	
Extrathyroidal extension (*n*=17)	0 (0%)	17 (3.60%)	
Composition			<0.001
Solid (*n*=454)	62 (54.39%)	392 (83.05%)	
Predominately solid(*n*=115)	39 (34.21%)	76 (16.10%)	
Predominately cystic (*n*=17)	13 (11.40%)	4 (0.85%)	
Echogenicity			<0.001^∗^
Hyperechogenicity (*n*=11)	3 (2.63%)	8 (1.69%)	
Isoechogenicity (*n*=136)	48 (42.11%)	88 (18.64%)	
Hypoechogenicity (*n*=246)	42 (36.84%)	204 (43.22%)	
Markedly hypoechoic(*n*=193)	21 (18.42%)	172 (36.44%)	
Echogenic foci			
punctate echogenic foci			<0.001^∗^
Microcalcification (*n*=335)	31 (27.19%)	304 (64.41%)	
Comet tail artifact (*n*=13)	11 (9.65%)	2 (0.42%)	
Punctate echogenic foci of undetermined significance (*n*=114)	21 (18.42%)	93 (19.70%)	
No punctate echogenic foci (*n*=124)	51 (44.74%)	73 (15.47%)	
Peripheral calcification			<0.001^∗^
With (*n*=36)	28 (24.56%)	8 (1.70%)	
Without (*n*=550)	86 (75.44%)	464 (98.31%)	
Macrocalcifications			0.707
With (*n*=81)	17 (14.91%)	64 (13.56%)	
Without (*n*=505)	97 (85.09%)	408 (86.44%)	

(The data were presented as the number, ∗ indicating a statistically significant difference).

The comparison of the C-TIRADS grading and pathological results is shown in **Table 3**. With grades 2 and 3 (i.e., –1 and 0), the pathological findings of the nodules were benign; malignant nodules were more common with grades 4C and 5 (i.e., 3, 4, and 5 points). Of the 586 nodules, 511 were diagnosed as TIRADS grade 4, and there were 87 benign nodules and 424 malignant ones as confirmed by the pathological results. Malignant nodules were mainly distributed among those with TIRADS grades 4C and 5 (especially grade 5; *P* < 0.001). The malignancy risk incidences calculated for TIRADS grades 4A, 4B, and 4C in the present study were higher than those given in the guidelines, while the malignancy risk incidences for TIRADS grades 2, 3, and 5 were consistent with those provided in the guidelines.

**Table 3:** C-TIRADS and pathological results.

The category in C-TIRADS	Score	The pathological results		The total number of cases	The calculated risk of malignant tumor
		Benign case	Malignant case		
1	–	–	–	–	–
2	-1	2	–	2	0
3	0	24	0	24	0
4A	1	30	8	38	21.05
4B	2	29	48	77	62.34
4C	3	20	204	224	91.07
	4	8	164	172	95.35
5	5	1	48	49	97.96
The total number of nodules	–	114	472	586	80.54

(The risks of malignancy for those with the grades of 4A, 4B, and 4C were higher than those recommended by the C-TIRADS guideline).

### The diagnostic performance of each parameter in SWE

**Figure 4:**
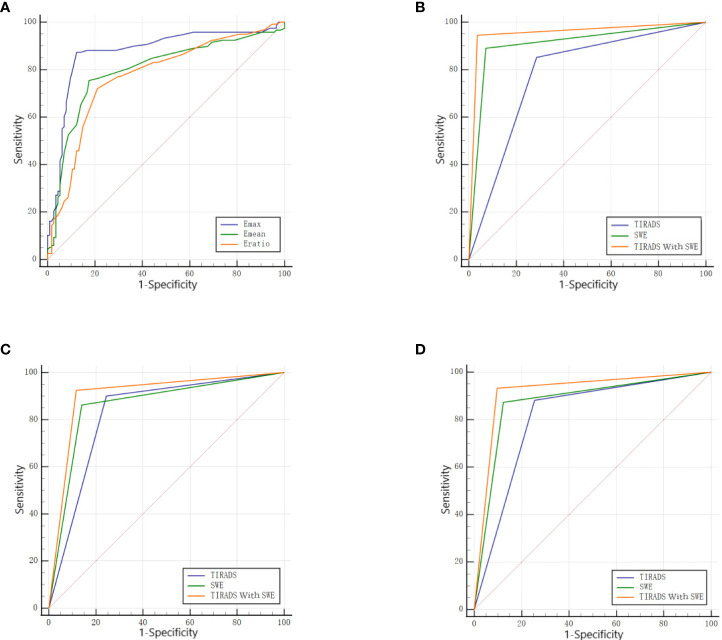
**(A)** The ROC of each parameter in SWE. **(B)** The ROC of the TIRADS alone, SWE alone, and the combination of the two in the D ≤ 10mm group. **(C)** The ROC of the TIRADS alone, SWE alone, and the combination of the two in the D>10mm group. **(D)** The ROC of the TIRADS alone, SWE alone, and the combination of the two in all nodules.

With the adoption of the pathological results as the gold standard, the ROC curves for each SWE parameter were constructed with the AUC comparison. The AUC of the Emax, Emean, and Eratio was 0.875, 0.798, and 0.772, respectively. The AUC of the Emax was higher than that of the Emean and Eratio (*P* < 0.001 in both). Therefore, the Emax was selected as the optimal parameter in combination with TIRADS. When Emax = 51 kPa was selected as the optimal cutoff point, 100 cases with benign nodules and 412 cases with malignant ones were correctly diagnosed. The diagnostic sensitivity, specificity, and accuracy of Emax were 87.29%, 87.72%, and 87.37%, respectively (**Table 4** and **Figure 4A**).

**Table 4:** Statistical analysis of using SWE parameters to differentiate benign and malignant thyroid lesions.

	The optimal cutoff point	The sensitivity(%)	The specificity(%)	95%CI	AUC
E_max_	51 kPa	87.29	87.72	0.846 - 0.901	0.875
E_mean_	29 kPa	75.42	82.46	0.763 - 0.829	0.798
E_ratio_	2.0	72.03	78.69	0.736 - 0.805	0.772

(CI, the confidential interval).

### The diagnostic performance of SWE, C-TIRADS, and the combination of SWE and C-TIRADS

In the D ≤10 mm group, the diagnostic sensitivity, specificity, and accuracy with the adoption of C-TIRADS were 85.16%, 71.43%, and 83.33%, respectively. The diagnostic sensitivity, specificity, and accuracy with the adoption of TIRADS combined with SWE were 94.51%, 96.43%, and 94.76%, respectively. The AUC of the combination of the two methods (0.955) was higher than that of C-TIRADS alone (0.783) or SWE alone (0.909), and the differences were statistically significant (*P* < 0.05 in both) (**Table 5** and **Figure 4B**).

**Table 5:** Analysis of the diagnostic value of TIRADS, SWE and their combination in thyroid nodules.

	AUC	The sensitivity (%)	The specificity (%)	The accuracy (%)	The positive predictive value (%)	The negative predictive value (%)
D ≤ 10mm						
TIRADS	0.783	85.16	71.43	83.33	95.09	42.55
SWE	0.909	89.01	92.86	89.52	98.78	56.52
The combination of TIRADS with SWE	0.955	94.51	96.43	94.76	99.42	72.97
D>10mm						
TI-RADS	0.828	90.00	75.58	86.70	92.55	69.15
SWE	0.861	86.21	86.05	86.17	95.42	64.91
The combination of TIRADS with SWE	0.904	92.41	88.37	91.49	96.40	77.55
In all nodules						
TI-RADS	0.813	88.14	74.56	85.50	93.48	60.28
SWE	0.875	87.29	87.72	87.37	96.71	62.50
The combination of TIRADS with SWE	0.918	93.22	90.35	92.66	97.56	76.30

(The sensitivity, specificity, accuracy, positive predictive value, and negative predictive value of patients in the present group were not further compared).

In the D >10 mm group, the diagnostic sensitivity, specificity, and accuracy with the adoption of C-TIRADS were 90.00%, 75.58%, and 86.70%, respectively. The diagnostic sensitivity, specificity, and accuracy with the adoption of TIRADS combined with SWE were 92.41%, 88.37%, and 91.49%, respectively. The AUC of the combination of the two methods (0.904) was higher than that of C-TIRADS alone (0.828) or SWE alone (0.861), and the differences were statistically significant (*P* < 0.05 in both) (**Table 5** and **Figure 4C**).

For all nodules, the diagnostic sensitivity, specificity, and accuracy with the adoption of C-TIRADS were 88.14%, 74.56%, and 85.50%, respectively. The diagnostic sensitivity, specificity, and accuracy with the adoption of TIRADS combined with SWE were 93.22%, 90.35%, and 92.66%, respectively. The AUC of the combination of the two methods (0.918) was higher than that of C-TIRADS alone (0.813) or SWE alone (0.875), and the differences were statistically significant (*P* < 0.05 in both) (**Table 5** and **Figure 4D**).

The DeLong test results showed that there was no statistical difference in AUC values of C-TIRADS, SWE and their combined diagnosis between D ≤ 10mm and D>10mm groups (ALL P > 0.05). The AUC increased from 0.783 to 0.955 in the D ≤10 mm group and from 0.828 to 0.904 in the D >10 mm group with the combination of SWE. The increase in the AUC with the combination of SWE was more obvious in the D ≤10 mm group than in the D >10 mm group.

## Discussion

Epidemiological investigations have shown that the incidence of thyroid malignancies has slowly increased over the past few years (12, 13). The thyroid, an organ close to the skin surface, is well suited for ultrasonography, but the accuracy of the diagnosis of thyroid nodules using only one grayscale ultrasonography sign is low. To improve the diagnostic accuracy, avoid economic hardship and psychological pressure for patients caused by overdiagnosis or overtreatment, and facilitate mutual communication between ultrasonographers and clinicians, researchers have established many kinds of TIRADS classifications applicable to the thyroid gland, inspired by the BIRADS classification for the breast. In 2020, the Ultrasound Medicine Branch of the Chinese Medical Association published the Chinese version of the TIRADS classification. The guidelines are based on the Chinese context with the adoption of a simple counting method to provide a detailed description of grayscale ultrasonography characteristics and are expected to solve the aforementioned problems (3).

In the present study, data from patients with thyroid nodules in a northeastern province of China were collected and graded according to C-TIRADS, and the sensitivity, specificity, and accuracy of C-TIRADS in identifying benign and malignant thyroid nodules were found to be 88.14%, 74.56%, and 85.50%, respectively. The diagnostic performance AUC was 0.813, which was similar to Qi’s results (14) and lower than Qiao et al.’s results (15). This might have been related to the proportion of micronodules and geographical factors. However, these studies demonstrated a high diagnostic performance of C-TIRADS in differentiating benign and malignant thyroid nodules. Zhu (16) and Qi (14) compared the diagnostic performance of C-TIRADS with other guidelines and showed that the AUC of C-TIRADS is higher than that of the other TIRADS guidelines, indicating that it has better diagnostic performance and might reduce the rate of unnecessary FNA.

Shear wave elastography is a technique that generates transverse shear waves in tissue and measures the shear wave velocity, which is then converted into Young’s modulus. The obtained Young’s modulus value can be used to evaluate the degree of stiffness of nodules. The stiffness of nodules is usually positively correlated with the risk of malignancy. Therefore, shear-wave elastography is an important tool for ultrasound diagnosis of thyroid nodules in clinical practice (17). Studies have not yet reached a consensus on the optimal parameters to distinguish between benign and malignant thyroid nodules or the cutoff points for the optimal parameters (Emax: 34.15–94.0 kPa, Emean: 26–85.2 kPa) (6–8). In the present study, the optimal Emax cutoff point was 51 kPa with an AUC of 0.875, making it superior to the Emean (0.788) and Eratio (0.772) as a parameter with optimal diagnostic efficacy (*P* < 0.05). These results were similar to those of Zhao et al. (6) The discrepancy between studies might be due to the differences in cancer subtypes. In the present study, the pathological type of all malignancies was papillary carcinoma, and there was a lack of non-papillary-carcinoma types such as follicular carcinoma and undifferentiated carcinoma. More prospective studies are needed in the future to include a proportional number of non-papillary carcinomas to obtain more consistent optimal parameters and cutoff points. The sensitivity, specificity, and accuracy of the Emax in the present study were 87.29%, 87.72%, and 87.37%, respectively, lower than those of previous studies (7). One possible reason might be that 14.91% of benign nodules were combined with Macrocalcifications, while Macrocalcifications tended to increase the nodule elasticity, resulting in false positivity. In addition, 16.95% were non-solid malignant nodules, when combined with cystic lesions in malignant nodules, which might have resulted in a decrease in stiffness and elasticity, which were easily misdiagnosed as benign ones. Some nodules were also complicated with diffused thyroid disease (e.g., nodular goiter or inflammatory thyroid disease) with lymphocytic infiltration and fibrosis, resulting in changes in the stiffness of the thyroid gland, making it difficult to distinguish the elasticity of the nodule from the thyroid parenchyma nearby. Due to these limitations, the elastography procedure should avoid measurement of macrocalcifications and cystic areas, and overdependence on elastography should be avoided for nodules with significant diffuse lesions in the background.

Many researchers have investigated whether the combination of SWE and TIRADS could improve the diagnostic efficacy for thyroid nodules, and achieved positive conclusions.

HANG (9) et al. found that the combination of TIRADS+SWE showed higher specificity (88.4% vs 73.6%) and positive predictive value (91.2% vs 83.2%) compared to TI-RADS alone. Zhang (10) et al. concluded that the combination of the ACR TIRADS and SWE Emax could improve diagnostic sensitivity and accuracy (94.2% Vs 81.4%, 90.7% vs 80.3%), the combination of the two methods in this study is similar to that in our study. Petersen M (11) et al. calculated that The addition of Elastography resulted in an increase of accuracy from 65.6% to 82.0% when using Kwak-tirads from 49.2% to 72.1% when using EU-TIRADS, indicated that the combination of TIRADS and SWE seem to be superior for the risk stratification of thyroid nodules at intermediate and high risk than each method alone.

At the same time, some studies have drawn different conclusions from this paper. A prospective study (18) in Italy analyzed Semiquantitative Strain Ratio (SRE) and Quantitative Shear Wave Elastography (SWE) in Association with TIRADS Classification,found that the combination of SRE+TIRADS and SRE+TIRADS+SWE obtained higher AUC values than TIRADS alone (0.85,0.82 vs 0.72). However, the addition of SWE didn’t increase the diagnostic capability of SRE+TIRADS combination. The SRE cut-off used is 1.92 and SWE is 37.5kPa in this study. Another study (19)found that the combined effect of K-TIRADS +SWE was not as good as that of K-TIRADS alone (AUC values were 0.72 and 0.77, respectively), where the optimal cut-off of SWE was 36.8kPa.

Differences in the epidemiology of diffuse thyroid lesions between regions and the composition of the pathological subtype of thyroid nodules included in each study may explain the differences in the above results. According to one epidemiological study (20), the prevalence of hyperthyroidism and hypothyroidism is higher in China than in Europe and North America. Long-term diffuse thyroid disease may lead to sclerosis and fibrosis of thyroid tissue, resulting in changes in thyroid echotexture and stiffness, and ultimately affect the choice of optimal SWE parameters and optimal cut-off from different studies.

According to a meta-analysis (21), SWE, as a shear-wave-based quantitative technique, has lower diagnostic accuracy than SE (Strain Elastography) and SRE (Strain Ratio Elastography). Meanwhile, it was found that the sensitivity of SE was higher than that of SWE, but the specificity was similar (22, 23). We believe that the reliability of the results of some articles is worth discussing. Some studies include SWE of different technologies (e.g., VTQ,VTIQ,ARFI, etc.), and the SWE-Acquisition and ROI placement processes prescribed for each technology are different. The parameters and optimal cut-off values used were not unified, so the heterogeneity between studies may be the source of bias. In fact, there is no agreement on the selection of cut-off points for SRE too (21), so each approach is not individually uniform. It is undeniable that the above methods are valuable for differentiating benign and malignant thyroid nodules (24–26). SWE has its own advantages. In the presence of diffuse thyroid lesions, SWE is less affected by the analysis of nodules, and the influence of precompression of SWE is better controlled than that of SE.

In the C-TIRADS guidelines, the panel pointed out that shear wave propagation velocity or Young’s modulus values measured by different instruments differ greatly in shear wave elastic imaging, and cannot be referred to each other (3). Different research methods and elastic imaging techniques limit the use of a single elastic value as a basis for differentiating benign and malignant thyroid nodules. SWE is more of a supplement to conventional grayscale ultrasound (27), providing more accurate and reliable information for the clinic, and then providing guidance for the selection of optimal treatment plan. At the same time, we hope that standardized guidelines will clarify the methodological issues related to elastography and improve the reproducibility of SWE.

Due to the too-short publication time of the C-TIRADS, relevant studies are still lacking. In the present study, the AUC of the combination of C-TIRADS and SWE for diagnosis was greater than that of C-TIRADS or SWE alone, confirming that SWE could also enhance the diagnostic performance of C-TIRADS for thyroid nodules. The enhancement was significant for the nodules in the D ≤10 mm group compared with those in the D >10 mm group. A possible reason might be that due to the small size of micro thyroid nodules, ultrasonography signs, such as lesion echogenicity, nature, and foci of calcification, tend to be poorly demonstrated and difficult to judge accurately, thus increasing the difficulty of the TIRADS grading. The present study also confirmed that the diagnostic AUC of the C-TIRADS in the D ≤10 mm group was lower than that in the D >10 mm group; the conclusions were similar to those obtained by Zhu et al. (16, 28) Moreover, the malignant nodules were all papillary carcinomas, which have fewer interstitial components, a harder texture, and a greater elasticity difference from the surrounding area. They were easy to diagnose with the adoption of SWE, and the diagnosis was not easily affected by the size of the nodules. The above results suggested that SWE and TIRADS are complementary, with TIRADS providing the morphological features of a nodule and SWE assessing the hardness. The combination of the two methods could improve the differentiation of benign and malignant thyroid nodules, especially for micronodules with unclear ultrasonography features.

There were several limitations in the present study. First, this was a retrospective study with the inclusion of only a few cases from one hospital, which was not representative of the Chinese population. Second, the image evaluation had its own limitations. The conventional ultrasonography and elastography of the nodules were conducted by one physician, and the assessment of ultrasound features and TIRADS classification based on the static images was conducted by three other physicians. The static images tended to cause inaccurate assessments of certain ultrasonography features; real-time dynamic images would have allowed for a more accurate assessment. Third, our hospital is a tertiary referral center, treating mainly oncological and other related diseases, with a higher incidence of thyroid cancer than other centers. The number of low-grade nodules was decreased, and the number of high-grade was nodules increased. These might have led to selection bias, affecting the diagnostic performance of the guidelines and reducing the diagnostic consistency. Fourth, the malignant nodules in the present study were all of a single pathological type (papillary carcinoma), making it difficult to assess the diagnostic performance of the above methods for other pathological types of malignant lesions. Finally, the intra- and interobserver variability were not analyzed. However, we interpreted, co-learned, and co-analyzed the guidelines before the beginning of the study for standardization purposes and to avoid reading bias. In summary, all of the abovementioned factors might have influenced the assessment of the diagnostic methods studied here.

## Conclusion

C-TIRADS is effective in assessing the risk of malignant thyroid nodules. The clinical application value of SWE parameter Emax was significantly higher than Emean and Eratio. SWE can be combined with C-TIRADS to improve its diagnostic value in the differential diagnosis of benign and malignant thyroid nodules, especially for nodules with D ≤ 10mm.

## Data availability statement

The original contributions presented in the study are included in the article/supplementary material. Further inquiries can be directed to the corresponding author.

## Ethics statement

The studies involving human participants were reviewed and approved by Harbin Medical University Cancer Hospital. The patients/participants provided their written informed consent to participate in this study.

## Author contributions

Conception and design of the research: X-QG and YM. Acquisition of data: X-SP, L-LW, H-XL, and X-LZ. Analysis and interpretation of the data: X-QG, YM, and X-SP. Statistical analysis: X-QG, YM, and X-SP. Obtaining financing: YL and L-LW. Writing of the manuscript: X-QG and YM. Critical revision of the manuscript for intellectual content: YL, X-LZ, and H-XL. All authors read and approved the final draft.

## Funding

Haiyan scientific research foundation of the Harbin Medical University Cancer Hospital (JJMS2021-02).

## Conflict of interest

The authors declare that the research was conducted in the absence of any commercial or financial relationships that could be construed as a potential conflict of interest.

## Publisher’s note

All claims expressed in this article are solely those of the authors and do not necessarily represent those of their affiliated organizations, or those of the publisher, the editors and the reviewers. Any product that may be evaluated in this article, or claim that may be made by its manufacturer, is not guaranteed or endorsed by the publisher.
